# A new sequence convergent to Euler–Mascheroni constant

**DOI:** 10.1186/s13660-018-1670-6

**Published:** 2018-04-05

**Authors:** Xu You, Di-Rong Chen

**Affiliations:** 1 Department of Mathematics and Physics, Beijing Institute of Petrochemical Technology, Beijing, P.R. China; 2 Department of Mathematics, Wuhan Textile University, Wuhan, P.R. China

**Keywords:** 11Y60, 41A25, 41A20, Euler–Mascheroni constant, Rate of convergence, Taylor’s formula, Harmonic sequence

## Abstract

In this paper, we provide a new sequence converging to the Euler–Mascheroni constant. Finally, we establish some inequalities for the Euler–Mascheroni constant by the new sequence.

## Introduction

The Euler–Mascheroni constant was first introduced by Leonhard Euler (1707–1783) in 1734 as the limit of the sequence

1.1 $$\begin{aligned} \gamma(n):=\sum_{m=1}^{n}\frac{1}{m} -\ln n. \end{aligned}$$

There are many famous unsolved problems about the nature of this constant (see, e.g., the survey papers or books of Brent and Zimmermann [1], Dence and Dence [2], Havil [3], and Lagarias [4]). For example, it is a long-standing open problem if the Euler–Mascheroni constant is a rational number. A good part of its mystery comes from the fact that the known algorithms converging to *γ* are not very fast, at least when they are compared to similar algorithms for *π* and *e*.

The sequence $(\gamma(n) )_{n\in\mathbb{N}}$ converges very slowly toward *γ*, like $(2n)^{-1}$. Up to now, many authors are preoccupied to improve its rate of convergence; see, for example, [2, 5–14] and references therein. We list some main results:

$$\begin{aligned} &\sum_{m=1}^{n}\frac{1}{m} -\ln \biggl(n+\frac{1}{2} \biggr)=\gamma+O\bigl(n^{-2}\bigr) \quad \mbox{(DeTemple [6]),} \\ &\sum_{m=1}^{n}\frac{1}{m} -\ln \frac{n^{3}+\frac{3}{2}n^{2}+\frac{227}{240}+\frac{107}{480}}{ n^{2}+n+\frac{97}{240}}=\gamma+O\bigl(n^{-6}\bigr) \quad\mbox{(Mortici [13]),} \\ & \begin{aligned} &\sum_{m=1}^{n}\frac{1}{m} -\ln \biggl( 1+\frac{1}{2n}+\frac{1}{24n^{2}}-\frac{1}{48n^{3}} +\frac{23}{5760n^{4}} \biggr)=\gamma+O\bigl(n^{-5}\bigr)\\ & \quad\mbox{(Chen and Mortici [5]).} \end{aligned} \end{aligned}$$

Recently, Mortici and Chen [14] provided a very interesting sequence

$$\begin{aligned} \nu(n)&=\sum_{m=1}^{n}\frac{1}{m} - \frac{1}{2}\ln \biggl(n^{2}+n+\frac{1}{3} \biggr) \\ &\quad{}- \biggl(\frac{-\frac{1}{180}}{ (n^{2}+n+\frac{1}{3} )^{2}} +\frac{\frac{8}{2835}}{ (n^{2}+n+\frac{1}{3} )^{3}} +\frac{\frac{5}{1512}}{ (n^{2}+n+\frac{1}{3} )^{4}} + \frac{\frac{592}{93\text{,}555}}{ (n^{2}+n+\frac{1}{3} )^{5}} \biggr) \end{aligned}$$

and proved that

1.2 $$\begin{aligned} \lim_{n\rightarrow\infty}n^{12} \bigl(\nu(n)-\gamma \bigr)=- \frac {796\text{,}801}{43\text{,}783\text{,}740}. \end{aligned}$$

Hence the rate of the convergence of the sequence $(\nu(n) )_{n\in\mathbb{N}}$ is $n^{-12}$.

Very recently, by inserting the continued fraction term into (1.1), Lu [9] introduced a class of sequences $(r_{k}(n) )_{n\in\mathbb{N}}$ (see Theorem 1) and showed that

1.3 $$\begin{aligned} &\frac{1}{72(n+1)^{3}}< \gamma-r_{2}(n)< \frac{1}{72n^{3}}, \end{aligned}$$

1.4 $$\begin{aligned} &\frac{1}{120(n+1)^{4}}< r_{3}(n)-\gamma< \frac{1}{120(n-1)^{4}}. \end{aligned}$$

In fact, Lu [9] also found $a_{4}$ without proof, and his works motivate our study. In this paper, starting from the well-known sequence $\gamma_{n}$, based on the early works of Mortici, DeTemple, and Lu, we provide some new classes of convergent sequences for the Euler–Mascheroni constant.

### Theorem 1

*For the Euler–Mascheroni constant*, *we have the following convergent sequence*:

$$\begin{aligned} r(n)=1+\frac{1}{2} +\cdots+\frac{1}{n}-\ln n-\ln \biggl(1+ \frac{a_{1}}{n} \biggr)-\ln \biggl(1+\frac{a_{2}}{n^{2}} \biggr)-\cdots, \end{aligned}$$

*where*

$$\begin{aligned} &a_{1}=\frac{1}{2},\qquad a_{2}=\frac{1}{24},\qquad a_{3}=-\frac{1}{24},\qquad a_{4}=\frac {143}{5760}, \\ &a_{5}=-\frac{1}{160}, \qquad a_{6}=-\frac{151}{290\text{,}304},\qquad a_{7}=-\frac{1}{896},\qquad \dots. \end{aligned} $$

*Let*

$$\begin{aligned} r_{k}(n):=\sum_{m=1}^{n} \frac{1}{m}-\ln n-\sum_{m=1}^{k}\ln \biggl(1+\frac {a_{m}}{n^{m}} \biggr). \end{aligned}$$

*For* $1\le k\le7$, *we have*

1.5 $$\begin{aligned} \lim_{n\rightarrow\infty}n^{k+2} \bigl(r_{k}(n)-\gamma \bigr)=C_{k}, \end{aligned}$$

*where*

$$\begin{aligned} &C_{1}=\frac{1}{24},\qquad C_{2}=-\frac{1}{24},\qquad C_{3}=\frac{143}{5760},\qquad C_{4}=-\frac{1}{160},\\ &C_{5}=-\frac{151}{290\text{,}304},\qquad C_{6}=-\frac{1}{896},\qquad C_{7}=\frac{109\text{,}793}{22\text{,}118\text{,}400},\qquad \dots. \end{aligned} $$

Furthermore, for $r_{2}(n)$ and $r_{3}(n)$, we also have the following inequalities.

### Theorem 2

*Let* $r_{2}(n)$ *and* $r_{3}(n)$ *be as in Theorem *1. *Then*

1.6 $$\begin{aligned} &\frac{1}{24}\frac{1}{(n+1)^{3}}< \gamma-r_{2}(n)< \frac{1}{24}\frac {1}{n^{3}}, \end{aligned}$$

1.7 $$\begin{aligned} &\frac{143}{5760}\frac{1}{(n+1)^{4}}< r_{3}(n)-\gamma< \frac{143}{5760}\frac {1}{n^{4}}. \end{aligned}$$

### Remark 1

Certainly, there are similar inequalities for $r_{k}(n)$ ($1\le k\le7$); we omit the details.

## Proof of Theorem 1

The following lemma gives a method for measuring the rate of convergence. This lemma was first used by Mortici [15, 16] for constructing asymptotic expansions or accelerating some convergences. For a proof and other details, see, for example, [16].

### Lemma 1

*If the sequence* $(x_{n})_{n\in\mathbb{N}}$ *converges to zero and there exists the limit*

2.1 $$\begin{aligned} \lim_{n\rightarrow+\infty}n^{s}(x_{n}-x_{n+1})=l \in[-\infty,+\infty] \end{aligned}$$

*with* $s>1$, *then there exists the limit*

2.2 $$\begin{aligned} \lim_{n\rightarrow+\infty}n^{s-1}x_{n}=\frac{l}{s-1}. \end{aligned}$$

We need to find the value $a_{1}\in\mathbb{R}$ that produces the most accurate approximation of the form

2.3 $$\begin{aligned} r_{1}(n)=\sum_{m=1}^{n} \frac{1}{m} -\ln n-\ln \biggl(1+\frac{a_{1}}{n} \biggr). \end{aligned}$$

To measure the accuracy of this approximation, we usually say that an approximation (2.3) is better as $r_{1}(n) -\gamma$ faster converges to zero. Clearly,

2.4 $$\begin{aligned} r_{1}(n)-r_{1}(n+1)=\ln \biggl(1+\frac{1}{n} \biggr)-\frac{1}{n+1}+\ln \biggl(1+\frac{a_{1}}{n+1} \biggr)-\ln \biggl(1+ \frac{a_{1}}{n} \biggr). \end{aligned}$$

Developing expression (2.4) into a power series expansion in $1/n$, we obtain

2.5 $$\begin{aligned} r_{1}(n)-r_{1}(n+1)= \biggl(\frac{1}{2} -a_{1} \biggr)\frac{1}{n^{2}}+ \biggl(-\frac{2}{3}+a_{1}+{a_{1}}^{2} \biggr)\frac{1}{n^{3}}+O \biggl(\frac{1}{n^{4}} \biggr). \end{aligned}$$

From Lemma 1 we see that the rate of convergence of the sequence $(r_{1}(n)-\gamma )_{n\in\mathbb{N}}$ is even higher as the value *s* satisfies (2.1). By Lemma 1 we have

(i) If $a_{1}\neq1/2$, then the rate of convergence of the $(r_{1}(n)-\gamma )_{n\in\mathbb{N}}$ is $n^{-2}$, since

$$\begin{aligned} \lim_{n\rightarrow\infty}n \bigl(r_{1}(n)-\gamma \bigr)= \frac{1}{2}-a_{1}\neq0. \end{aligned}$$

(ii) If $a_{1}= 1/2$, then from (2.5) we have

$$\begin{aligned} r_{1}(n)-r_{1}(n+1)=\frac{1}{12}\frac{1}{n^{3}}+O \biggl(\frac{1}{n^{4}} \biggr). \end{aligned}$$

Hence the rate of convergence of the $(r_{1}(n)-\gamma )_{n\in\mathbb{N}}$ is $n^{-3}$, since

$$\begin{aligned} \lim_{n\rightarrow\infty}n^{3} \bigl(r_{1}(n)-\gamma \bigr)=\frac{1}{24}:=C_{1}. \end{aligned}$$

We also observe that the fastest possible sequence $(r_{1}(n) )_{n\in\mathbb{N}}$ is obtained only for $a_{1}=1/2$.

We repeat our approach to determine $a_{1}$ to $a_{7}$ step by step. In fact, we can easily compute $a_{k}$, $k\leq15$, by the *Mathematica* software. In this paper, we use the *Mathematica* software to manipulate symbolic computations.

Let

2.6 $$\begin{aligned} r_{k}(n):=\sum_{m=1}^{n} \frac{1}{m}-\ln n-\sum_{m=1}^{k}\ln \biggl(1+\frac {a_{m}}{n^{m}} \biggr). \end{aligned}$$

Then

2.7 $$\begin{aligned} &r_{k}(n)-r_{k}(n+1) \\ &\quad =\ln \biggl(1+\frac{1}{n} \biggr)-\frac{1}{n+1}+\sum_{m=1}^{k}\ln \biggl(1+\frac{a_{m}}{(n+1)^{m}} \biggr)-\sum_{m=1}^{k} \ln \biggl(1+\frac{a_{m}}{n^{m}} \biggr). \end{aligned}$$

Hence the key step is to expand $r_{k}(n)-r_{k}(n+1)$ into power series in $1/n$. Here we use some examples to explain our method.

*Step 1*: For example, given $a_{1}$ to $a_{4}$, find $a_{5}$. Define

$$\begin{aligned} r_{5}(n):=\sum_{m=1}^{n} \frac{1}{m}-\ln n-\sum_{m=1}^{5}\ln \biggl(1+\frac {a_{m}}{n^{m}} \biggr). \end{aligned}$$

By using the *Mathematica* software (the *Mathematica Program* is very similar to that given further in Remark 2; however, it has the parameter $a_{8}$) we obtain

2.8 $$\begin{aligned} r_{5}(n)-r_{5}(n+1)= \biggl(-\frac{1}{32}-5a_{5} \biggr)\frac{1}{n^{6}}+ \biggl(\frac{4385}{48\text{,}384}+15a_{5} \biggr) \frac{1}{n^{7}}+O \biggl(\frac {1}{n^{8}} \biggr). \end{aligned}$$

The fastest possible sequence $(r_{5}(n) )_{n\in\mathbb{N}}$ is obtained only for $a_{5}=-\frac{1}{160}$. At the same time, it follows from (2.8) that

2.9 $$\begin{aligned} r_{5}(n)-r_{5}(n+1)=-\frac{151}{48\text{,}384}\frac{1}{n^{7}} +O \biggl(\frac {1}{n^{8}} \biggr). \end{aligned}$$

The rate of convergence of $(r_{8}(n)-\gamma )_{n\in\mathbb{N}}$ is $n^{-7}$, since

$$\begin{aligned} \lim_{n\rightarrow\infty}n^{7} \bigl(r_{5}(n)-\gamma \bigr)=-\frac{151}{290\text{,}304}:=C_{5}. \end{aligned}$$

We can use this approach to find $a_{k}$ ($1\le k\le15$). From the computations we may the conjecture $a_{n+1}=C_{n}$, $n\geq1$. Now, let us check it carefully.

*Step 2*: Check $a_{6}=-\frac{151}{290\text{,}304}$ to $a_{7}=-\frac{1}{896}$.

Let $a_{1},\dots, a_{6}$, and $r_{6}(n)$ be defined as in Theorem 1. Applying the *Mathematica* software, we obtain

2.10 $$\begin{aligned} r_{6}(n)-r_{6}(n+1)=-\frac{1}{128}\frac{1}{n^{8}}+O \biggl(\frac{1}{n^{9}} \biggr). \end{aligned}$$

The rate of convergence of $(r_{6}(n)-\gamma )_{n\in\mathbb{N}}$ is $n^{-8}$, since

$$\begin{aligned} \lim_{n\rightarrow\infty}n^{8} \bigl(r_{6}(n)-\gamma \bigr)=-\frac{1}{896}:=C_{6}. \end{aligned}$$

Finally, we check that $a_{7}=-\frac{1}{896}$:

2.11 $$\begin{aligned} r_{7}(n)-r_{7}(n+1)= \biggl(-\frac{1}{128}-7a_{7} \biggr)\frac{1}{n^{8}} + \biggl(\frac{196\text{,}193}{2\text{,}764\text{,}800}+28a_{7} \biggr) \frac{1}{n^{9}}+O \biggl(\frac {1}{n^{10}} \biggr). \end{aligned}$$

Since $a_{7}=-\frac{1}{896}$ and

$$\begin{aligned} \lim_{n\rightarrow\infty}n^{9} \bigl(r_{7}(n)-\gamma \bigr)= \frac{109\text{,}793}{22\text{,}118\text{,}400}:=C_{7}, \end{aligned}$$

the rate of convergence of the $(r_{7}(n)-\gamma )_{n\in\mathbb{N}}$ is $n^{-9}$.

This completes the proof of Theorem 1.

### Remark 2

From the computations we can guess that $a_{n+1}=C_{n}$, $n\geq1$. It is a very interesting problem to prove this. However, it seems impossible by the provided method.

## Proof of Theorem 2

Before we prove Theorem 2, let us give a simple inequality, which follows from the Hermite–Hadamard inequality and plays an important role in the proof.

### Lemma 2

*Let* *f* *be a twice continuously differentiable function*. *If* $f''(x)>0$, *then*

3.1 $$\begin{aligned} \int_{a}^{a+1}f(x) \,dx > f(a+1/2). \end{aligned}$$

By $P_{k}(x)$ we denote polynomials of degree *k* in *x* such that all its nonzero coefficients are positive; it may be different at each occurrence.

Let us prove Theorem 2. Noting that $r_{2}(\infty)=0$, we easily see that

3.2 $$\begin{aligned} \gamma-r_{2}(n)=\sum_{m=n}^{\infty} \bigl(r_{2}(m+1)-r_{2}(m) \bigr) =\sum _{m=n}^{\infty}f(m), \end{aligned}$$

where

$$\begin{aligned} f(m)&=\frac{1}{m+1}-\ln \biggl(1+\frac{1}{m} \biggr)-\ln \biggl(1+ \frac {a_{1}}{m+1} \biggr)-\ln \biggl(1+\frac{a_{2}}{(m+1)^{2}} \biggr)\\ &\quad {}+\ln \biggl(1+ \frac{a_{1}}{m} \biggr)+\ln \biggl(1+\frac{a_{2}}{m^{2}} \biggr). \end{aligned} $$

Let $D_{1}=1/2$. By using the *Mathematica* software we have

$$\begin{aligned} &-f'(x)-D_{1}\frac{1}{(x+1)^{5}}\\ &\quad =\frac {300+2739x+11\text{,}434x^{2}+24\text{,}870x^{3}+28\text{,}314x^{4}+15\text{,}936x^{5}+3472x^{6}}{ 2x(1+x)^{5}(1+2x)(3+2x)(1+24x^{2})(25+48x+24x^{2})}>0 \end{aligned}$$

and

$$\begin{aligned} & -f'(x)-D_{1}\frac{1}{(x+\frac{1}{2})^{5}}\\ &\quad =-\frac{P_{6}(x)(x-1)+151\text{,}085}{2x^{5} (1 +x)^{2} (1 + 2x) (3 + 2x) (1 + 24x^{2}) (25 + 48x + 24 x^{2})}< 0. \end{aligned}$$

Hence, we get the following inequalities for $x\ge1$:

3.3 $$\begin{aligned} D_{1}\frac{1}{(x+1)^{5}}< -f'(x)< D_{1} \frac{1}{(x+\frac{1}{2})^{5}}. \end{aligned}$$

Since $f(\infty)=0$, from the right-hand side of (3.3) and Lemma 2 we get

3.4 $$\begin{aligned} f(m)&= - \int_{m}^{\infty}f'(x) \,dx \le D_{1} \int_{m}^{\infty}\biggl( x+\frac{1}{2} \biggr)^{-5} \,dx \\ &=\frac{D_{1}}{4}\biggl(m+\frac{1}{2}\biggr)^{-4} \le \frac{D_{1}}{4} \int _{m}^{m+1}x^{-4} \,dx. \end{aligned}$$

From (3.1) and (3.4) we obtain

3.5 $$\begin{aligned} \gamma-r_{2}(n)&\le \sum_{m=n}^{\infty} \frac{D_{1}}{4} \int_{m}^{m+1}x^{-4} \,dx \\ &=\frac{D_{1}}{4} \int_{n}^{\infty}x^{-4} \,dx=\frac{D_{1}}{12} \frac {1}{n^{3}}. \end{aligned}$$

Similarly, we also have

$$\begin{aligned} f(m)&= - \int_{m}^{\infty}f'(x) \,dx \ge D_{1} \int_{m}^{\infty}(x+1)^{-5} \,dx \\ &=\frac{D_{1}}{4}(m+1)^{-4}\ge\frac{D_{1}}{4} \int_{m+1}^{m+2}x^{-4} \,dx \end{aligned}$$

and

3.6 $$ \begin{aligned}[b] \gamma-r_{2}(n)&\ge \sum_{m=n}^{\infty} \frac{D_{1}}{4} \int _{m+1}^{m+2}x^{-4} \,dx \\ &=\frac{D_{1}}{4} \int_{n+1}^{\infty}x^{-4} \,dx=\frac{D_{1}}{12} \frac {1}{(n+1)^{3}}. \end{aligned} $$

Combining (3.5) and (3.6) completes the proof of (1.6).

Noting that $r_{3}(\infty)=0$, we easily deduce

3.7 $$\begin{aligned} r_{3}(n)-\gamma=\sum_{m=n}^{\infty} \bigl(r_{3}(m)-r_{3}(m+1) \bigr)=\sum _{m=n}^{\infty}g(m), \end{aligned}$$

where

$$g(m)=r_{3}(m)-r_{3}(m+1). $$

Let $D_{2}=\frac{143}{288}$. By using the *Mathematica* software we have

$$\begin{aligned} &-g'(x)-D_{2}\frac{1}{(x+1)^{6}}\\ &\quad =\frac{P_{12}(x)}{288 n (1 + n)^{6} (1 + 2 n) (3 + 2 n) (1 + 24 n^{2}) (25 + 48 n +24 n^{2}) (-1 + 24 n^{3})P_{3}(x)}>0 \end{aligned}$$

and

$$\begin{aligned} &-g'(x)-D_{2}\frac{1}{(x+\frac{1}{2})^{6}}\\ &\quad =-\frac{P_{12}(x)(x-4)+2\text{,}052\text{,}948\text{,}001\text{,}087\text{,}775 }{9 x (1 + x)^{2} (1 + 2 x)^{6} (3 + 2 x) (1 + 24 x^{2}) (25 + 48 x + 24 x^{2}) (-1 + 24 x^{3})P_{3}(x)}< 0. \end{aligned}$$

Hence, for $x\ge4$,

3.8 $$\begin{aligned} D_{2}\frac{1}{(n+1)^{6}}< -g'(x)< D_{2} \frac{1}{(x+\frac{1}{2})^{6}}. \end{aligned}$$

Since $g(\infty)=0$, by (3.8) we get

3.9 $$\begin{aligned} g(m)&= - \int_{m}^{\infty}g'(x) \,dx \le D_{2} \int_{m}^{\infty} \biggl(x+\frac{1}{2} \biggr)^{-6} \,dx \\ &=\frac{D_{2}}{5} \biggl(m+\frac{1}{2} \biggr)^{-5} \le \frac{D_{2}}{5} \int_{m}^{m+1}x^{-5} \,dx. \end{aligned}$$

It follows from (3.7), (3.9), and Lemma 2 that

3.10 $$\begin{aligned} r_{3}(n)-\gamma&\le \sum_{m=n}^{\infty} \frac{D_{2}}{5} \int _{m}^{m+1}x^{-5} \,dx \\ &=\frac{D_{2}}{5} \int_{n}^{\infty}x^{-5} \,dx=\frac{D_{2}}{20} \frac {1}{n^{4}}. \end{aligned}$$

Finally,

$$\begin{aligned} g(m)&= - \int_{m}^{\infty}g'(x) \,dx \ge D_{2} \int_{m}^{\infty}(x+1)^{-6} \,dx \\ &=\frac{D_{2}}{5}(m+1)^{-5}\ge\frac{D_{2}}{5} \int_{m+1}^{m+2}x^{-5} \,dx \end{aligned}$$

and

3.11 $$\begin{aligned} r_{3}(n)-\gamma&\ge \sum_{m=n}^{\infty} \frac{D_{2}}{5} \int _{m+1}^{m+2}x^{-5} \,dx \\ &=\frac{D_{2}}{5} \int_{n+1}^{\infty}x^{-5} \,dx =\frac{D_{2}}{20} \frac{1}{(n+1)^{4}}. \end{aligned}$$

Combining (3.10) and (3.11) completes the proof of (1.7).
